# The first reported case of clicking larynx syndrome complicating thyroidectomy due to papillary thyroid cancer

**DOI:** 10.1016/j.ijscr.2023.108443

**Published:** 2023-06-29

**Authors:** Yumna Njoum, Amal Obeid, Tawfiq AbuKeshek, Mohammed Maree

**Affiliations:** a Faculty of Medicine, Al-Quds University, Jerusalem, Palestine; b Department of Surgery, Al-Makassed Hospital, Jerusalem, Palestine; c Department of Radiology, Al-Makassed Hospital, Jerusalem, Palestine

**Keywords:** Clicking larynx, Papillary thyroid Cancer, Thyroidectomy

## Abstract

**Introduction and importance:**

When the superior cornu or the top edge of the thyroid cartilage rubs against the hyoid, or when these structures come to rub against the cervical spine, Clicking Larynx Syndrome (CLS) occurs. Which is a very rare disorder in that only less than 20 cases are reported in the literature. Patients seldom ever mention past laryngeal injuries. The cause of the accompanying pain when present is yet unknown. Gold standard management appears to be thyroplastic surgery in which the structures responsible for the clicking sounds are removed or reduction of the size of the large horn of the hyoid bone.

**Case presentation:**

Herein, we present a 42-year-old male patient with a history of papillary thyroid microcarcinoma treated with left thyroidectomy presented with a spontaneous continuous painless clicking noise and abnormal clicking movement of the larynx.

**Clinical discussion:**

CLS is a very rare condition with a very limited number of cases reported worldwide, most reported cases revealed abnormal laryngeal structural anatomy. However, our patient had normal laryngeal structures where multiple diagnostic tools (i.e.: Computed tomography, laryngoscopy) failed to disclose causative abnormality to explain his symptoms, nor literature could reveal any previously reported similar causes or explain the causative relationship between our patient's history of thyroid malignancy or thyroidectomy with his condition.

**Conclusion:**

It is crucial to explain to patients with mild CLS that these clicking noises are safe and to provide them with information on the best possible case-dependent treatments to avoid the usually associated anxiety and psychological stress. Further observations and research are needed to analyze the association between thyroid malignancy, thyroidectomy and CLS.

## Highlights

• CLS is a rare disorder that only less than 20 cases are reported in the literature.
• CLS Presents when clicking sound originates upon swallowing, movement, palpation or spontaneously.
• Main causes are displaced superior cornu or short distance between thyroid cartilage and hyoid bone.
• Swallowing laryngeal Computed Tomography scanning is the gold standard diagnosis.

## 1 Introduction

CLS is noticed when a clicking sound is heard either upon movement of the neck, swallowing, or palpation or in even rare instances, as our patient presented: it occurs spontaneously (1). It is a very rare condition and is not well known to the general population or medical practitioners which, when present, can cause anxiety and psychological stress to the patient even in the absence of a serious or life-threatening medical condition warranting treatment (1).

The first ‘clicking larynx’ was described by Counter in 1978 (2) with no more than 20 cases reported in the literature (3). Although not fully explained, it’s usually attributed to an abnormally shortened distance connecting the hyoid bone with the thyroid cartilage (3). The Scarce data available showed a displaced location of the superior cornu of the thyroid cartilage or a short distance between the thyroid cartilage and hyoid bone to be the most common causes of clicking larynx. Most of which reported previous neck trauma (4). But after a thorough review of the literature, a history of thyroidectomy or thyroid malignancy as in our patient has never been reported before in any patient with CLS.

## 2 Case presentation

We present a case of a 42-year-old-male with a history of papillary thyroid cancer 3 years prior to the presentation that was treated by left hemithyroidectomy, presented complaining of shortness of breath, orthopnea, paroxysmal nocturnal dyspnea, left-sided neck discomfort, and generalized weakness. he denied dysphagia, odynophagia, or dysphonia, He also denied symptoms of cold intolerance, weight gain, or decreased appetite, nor history of heat intolerance, sweating, or irritability. He was noticed to have abnormal repetitive movement of his throat and a clicking sound that he admitted that he first noticed a few days after his thyroidectomy surgery that had a smooth post-operative course without complications. Clicking sounds occurred all the time with no provoking factors like moving his neck, swallowing, or speaking, he has no history of neck pain or previous similar episodes during childhood or before his malignancy.

A neck examination showed a well-healed thyroidectomy scar with no visible masses. There was an abnormal movement of thyroid cartilage associated with a clicking noise heard continuously not related to neck movement, swallowing, or talking and not associated with stridor or abnormal breathing sounds. Palpation of the neck did not cause any tenderness, neither the thyroid gland nor cervical lymph nodes were palpable. Carotid arteries were palpable bilaterally with regular pulse and auscultated. Oral examination revealed clicking movement of the tongue and throat structures. no enlarged tonsils or visible abnormalities were appreciated. Cranial nerves were intact with no focal neurological deficits.

**Fig. 1:**
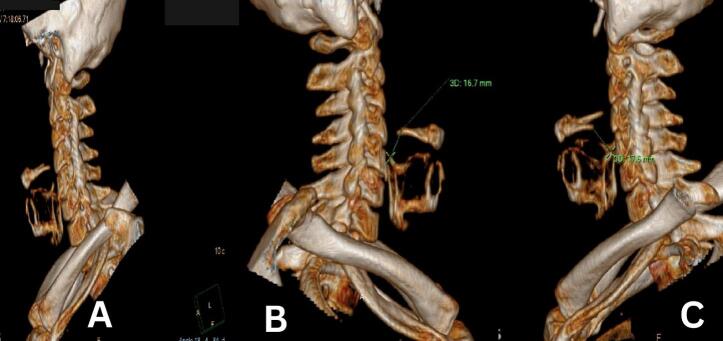
3D neck scan. General view (A.) right (B.) and left side (C.) view of the neck with measurement shows a normal distance between the superior cornu of the thyroid cartilage and the lower surface of the greater cornu of the hyoid bone.

Laboratory investigations showed normal complete blood count and thyroid function test levels, Neck ultrasound showed a right lobe of normal size and shape with no nodules, lesions, or lymph nodes visualized. A 9 × 6 mm left thyroid remnant is noted. He also underwent flexible fiberoptic laryngoscopy that showed no abnormal findings so a neck CT scan with 0.6-mm thick sections and 3D reconstruction (Fig. 1) showed normal hyoid bone size and normal distance between the superior cornu of the thyroid cartilage and the lower surface of the greater cornu of the hyoid bone. (On the right side: sagittal plane 16.7 mm. On the left side: sagittal plane 17.6 mm) remnant of thyroid tissue in the left thyroid lobe, a normal right thyroid lobe, with no evidence of neck masses, obvious tumor recurrence or structural cause explaining his clicking larynx (Fig. 1).

**Fig. 2:**
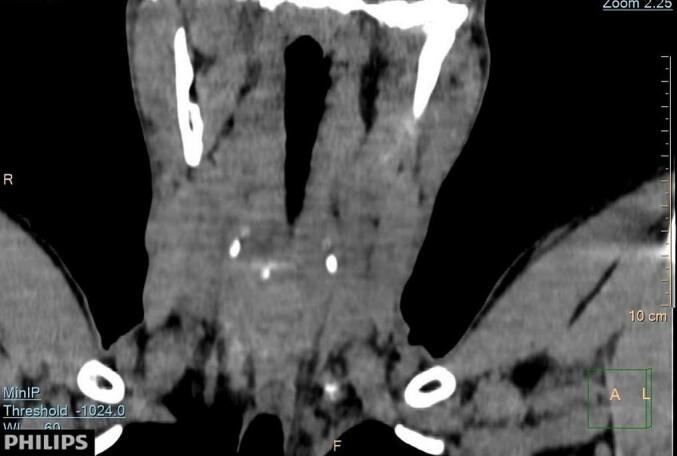
Image of coronal oblique Neck CT shows bilateral moderate ossification of triticeal cartilage.

Moderate ossification of triticeal cartilage bilaterally was noted (Fig. 2).

The patient was discussed regarding his condition and albeit opting for the conservative option, he was nevertheless relieved upon knowing his symptoms are not caused by malignancy recurrence or significant medical disease so he was scheduled for his regular oncology follow-up. The patient was phone called 7 months after diagnosis, he stated having the same complaint with no resolution of his condition that started 3 years before diagnosis. The patient is also cancer free and on regular oncology follow-ups.

## 3 Discussion

This rare entity of CLS remains highly intriguing with a very limited number of cases that could not cross the twenties border of the reported cases number which despite being a very small number, showed that most reported patients were females indicating young female predominance (3) unlike our 42-year-old male patient. The etiology is not well established but is thought to be due to an abnormally shortened distance connecting the hyoid bone with the thyroid cartilage and a displaced location of the superior cornu of the thyroid cartilage (3). While other reported cases were caused by cervical spine impingement by the greater horn of hyoid elongation or bulk and superior cornu of thyroid cartilage with hyoid bone friction caused by medial displacement of the superior cornu of thyroid cartilage (1,4) none of which explained the painless spontaneous continuous condition of our male patient with a history of thyroid malignancy and thyroidectomy, which to our best knowledge, could not be found reported before, making this association of thyroid malignancy, thyroidectomy and post-surgical CLS a question that deserves to be answered upon observation of thyroidectomy patients. A clicking larynx is a big challenge for surgeons to understand, diagnose and manage in the absence of clear guidelines. Diagnosis starts when patients present complaining of a clicking sound or sensation which can also be associated with pain. Physical examination is crucial for the assessment of the clicking location and the presence of associated findings which may help narrow the differential diagnosis (5), but swallowing laryngeal computed tomography (CT) scanning remains the modality of choice (6). Laryngeal endoscopy, cervical CT, and videofluoroscopic swallowing study (VFSS) are imaging techniques that can detect laryngeal clicks. Hard tissues, such as the hyoid bone and styloid process, cannot be examined with sufficient accuracy in VFSS because of inadequate image quality. While only the laryngopharynx's lumen may be evaluated during an endoscopy. Cervical CT provides a thorough picture of the entire neck, the distances of the anatomical references in the CT scan are very important for the diagnosis and the surgical plan for treatment. The mean distance between the superior thyroid cornua and the hyoid bone in women of 8.65 ± 4.47 mm (*n* = 100, right side 8.96 ± 4.48, and left side 8.20 ± 4.47 mm) (7).

Although it is challenging to detect dynamic structural changes, these difficulties can be solved by swallowing CT and swallowing virtual reality (swallowing VR), particularly when swallowing or movement-induced clicking is present (6).

Surgical correction remains the treatment of choice in cases of anatomical and structural disease. However, our case could not reveal any structural abnormality along with the lack of understandable etiology and management guidelines makes diagnosis options very limited.

## 4 Conclusion

CLS is a very rare etiology with less than 20 cases reported in the literature. It’s a clicking noise and abnormal repetitive movement of the larynx that can be explained by various anatomical etiologies. However, the literature never showed a correlation between neck and thyroid malignancy or thyroidectomy and the development of this rare entity in the absence of obvious laryngeal structural abnormality as witnessed in our patient. This correlation requires further observation and research to detect a causative explanation. However, after our experience, a CLS could complicate thyroid malignancy and thyroidectomy patients.

## Ethical approval

This case report is exempt from ethical approval in our institute. As a case report, it only required a written informed consent from the patient himself.

## Funding

There is no source of funding.

## Authors’ contribution

Yumna Njoum: Literature review and manuscript preparation.

Amal Obeid: Literature review and manuscript preparation.

Tawfiq AbuKeshek: Radiology part and diagnostics.

Mohammed Maree: Manuscript review and editing

## Guarantor

Dr. Mohammad Maree, Email: mohammedmaree1983@gmail.com.

## Informed consent

Written informed consent was obtained from the patient for the publication of this case report and accompanying images. A copy of the written consent is available for review by the Editor-in-Chief of this journal on request.

## Patient’s perspectives on the treatments they received

I 'm very relieved upon understanding more about the benign nature of my condition after what I 've been through and my history of thyroid cancer, I was nervous, stressed, and afraid it could be caused by malignancy recurrence or significant medical disease. Thus, I chose conservative management and no further treatment for my clicking larynx.

## Conflict of interest statement

There are no conflicts of interest.
